# Supplementary material for: Benchmarking multi-omics integration algorithms across single-cell RNA and ATAC data
Source: Brief Bioinform. 2024 Mar 16;25(2):bbae095. doi: 10.1093/bib/bbae095 (PMC10944570; doi:10.1093/bib/bbae095)
Supplement: Supplementary_figures_bbae095 [file supplementary_figures_bbae095.docx]

# **Supplementary Figures**


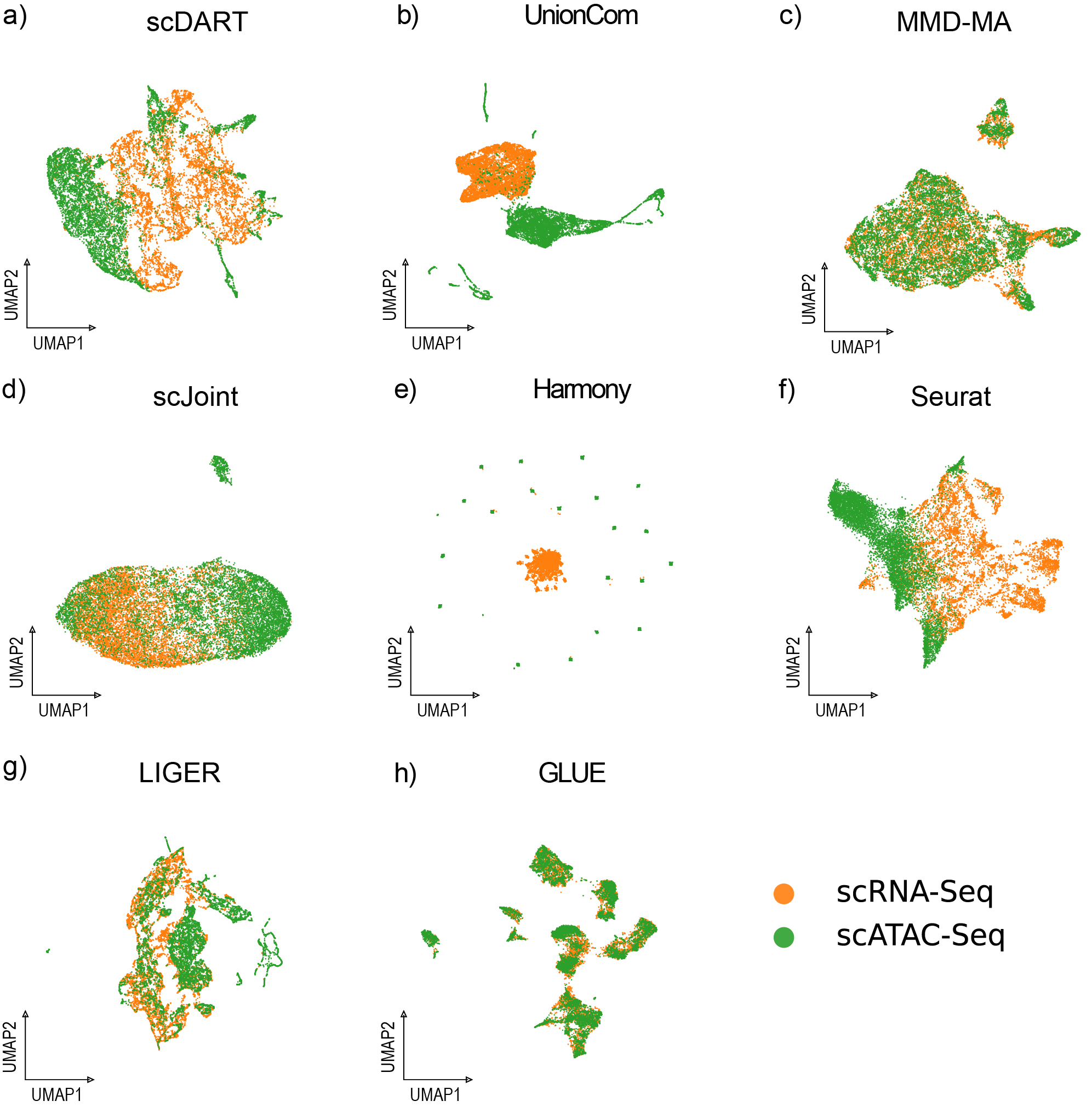


Fig. S1 | UMAP visualizations of the integrated cell embedding for Dataset-U, colored by omics types. Orange stands for scRNA-seq, and green stands for scATAC-seq.


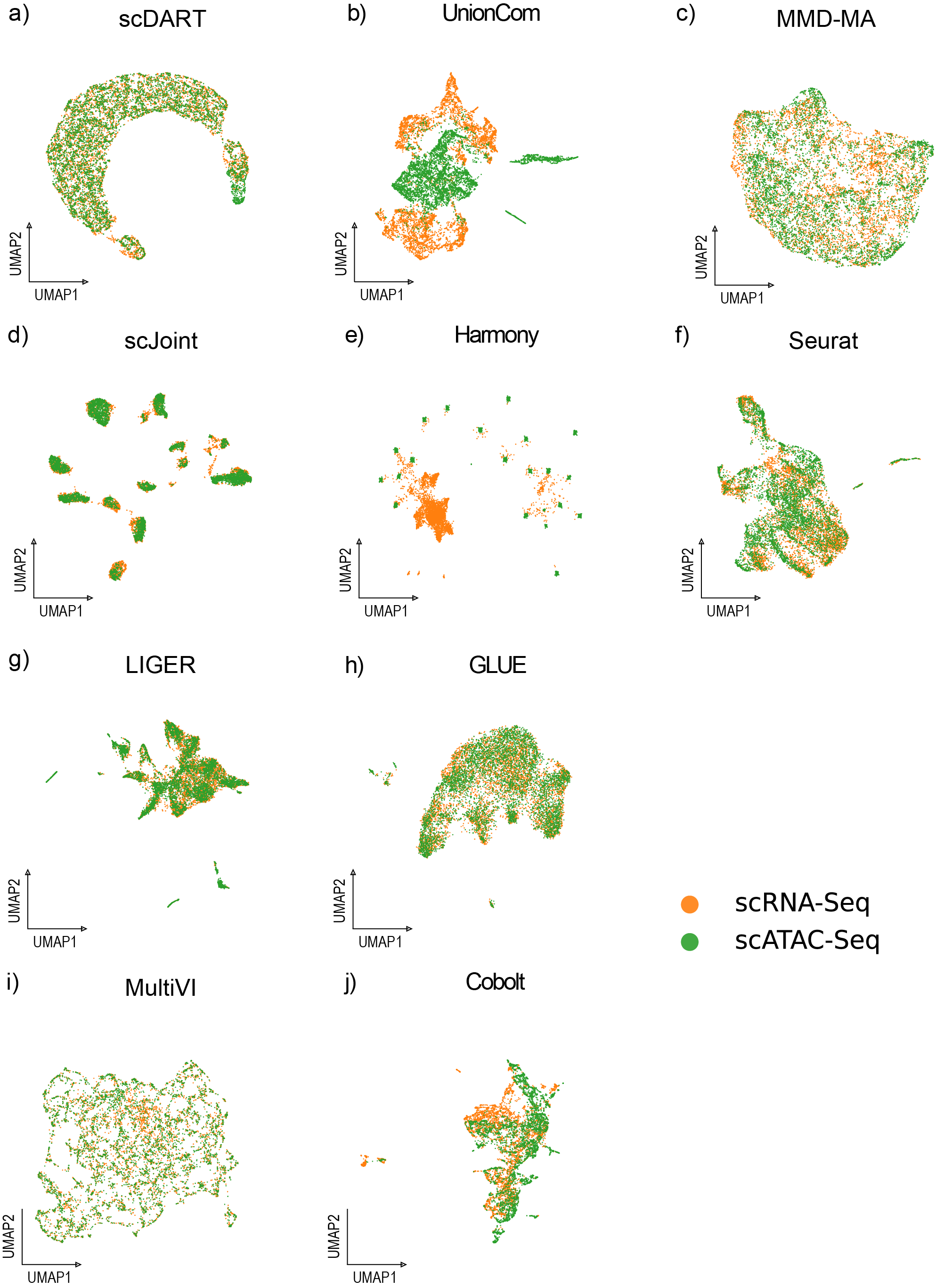


Fig. S2 | UMAP visualizations of the integrated cell embedding for Dataset-P, colored by omics types. Orange stands for scRNA-seq, and green stands for scATAC-seq. As the latent distributions of two omics were not separately provided by scMVP and MOFA+, this diagram for these two methods were not shown here.


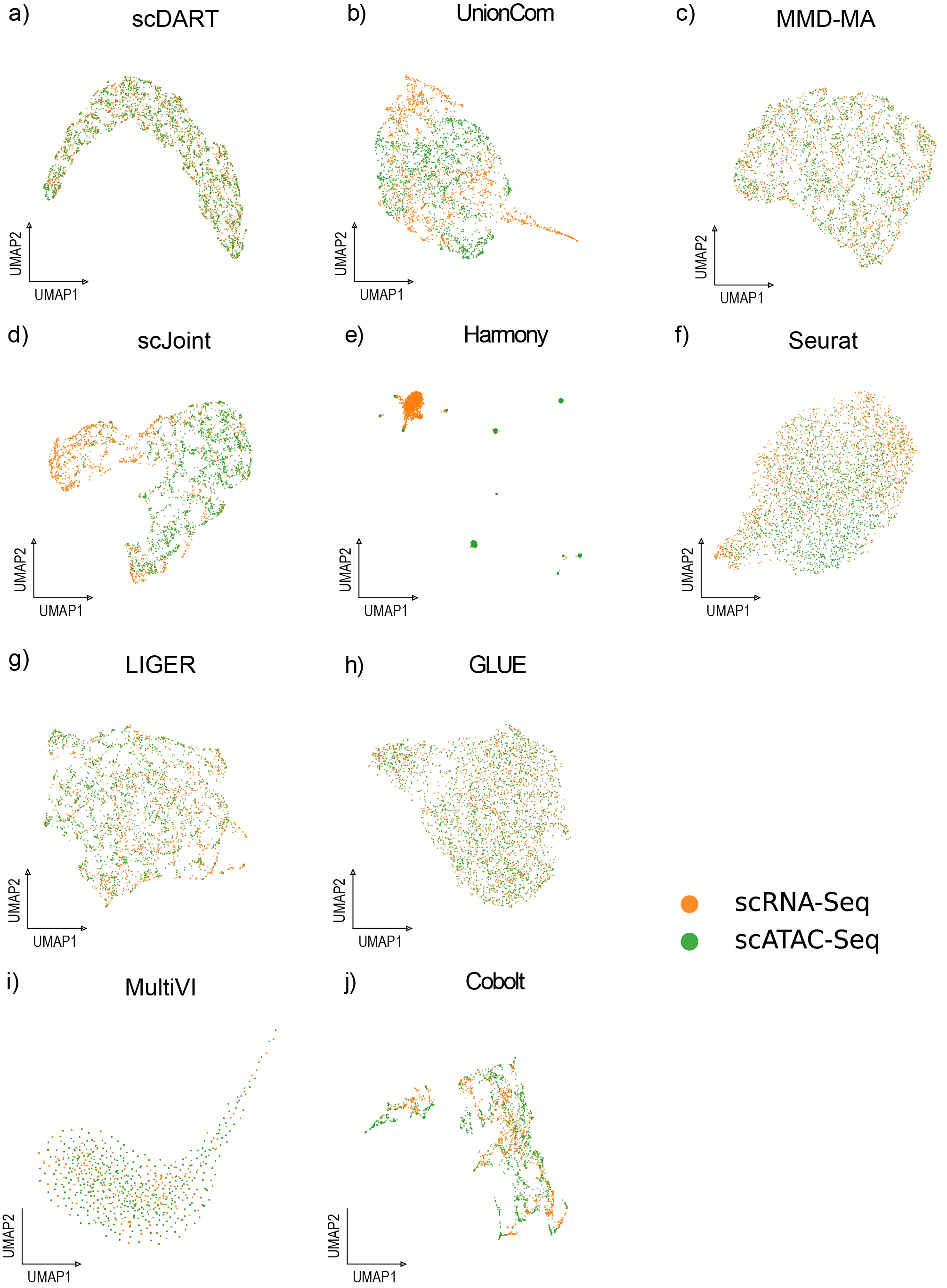


Fig. S3 | UMAP visualizations of the integrated cell embedding for Dataset-T, colored by omics types. Orange stands for scRNA-seq, and green stands for scATAC-seq. As the latent distributions of two omics were not separately provided by scMVP and MOFA+, this diagram for these two methods were not shown here.


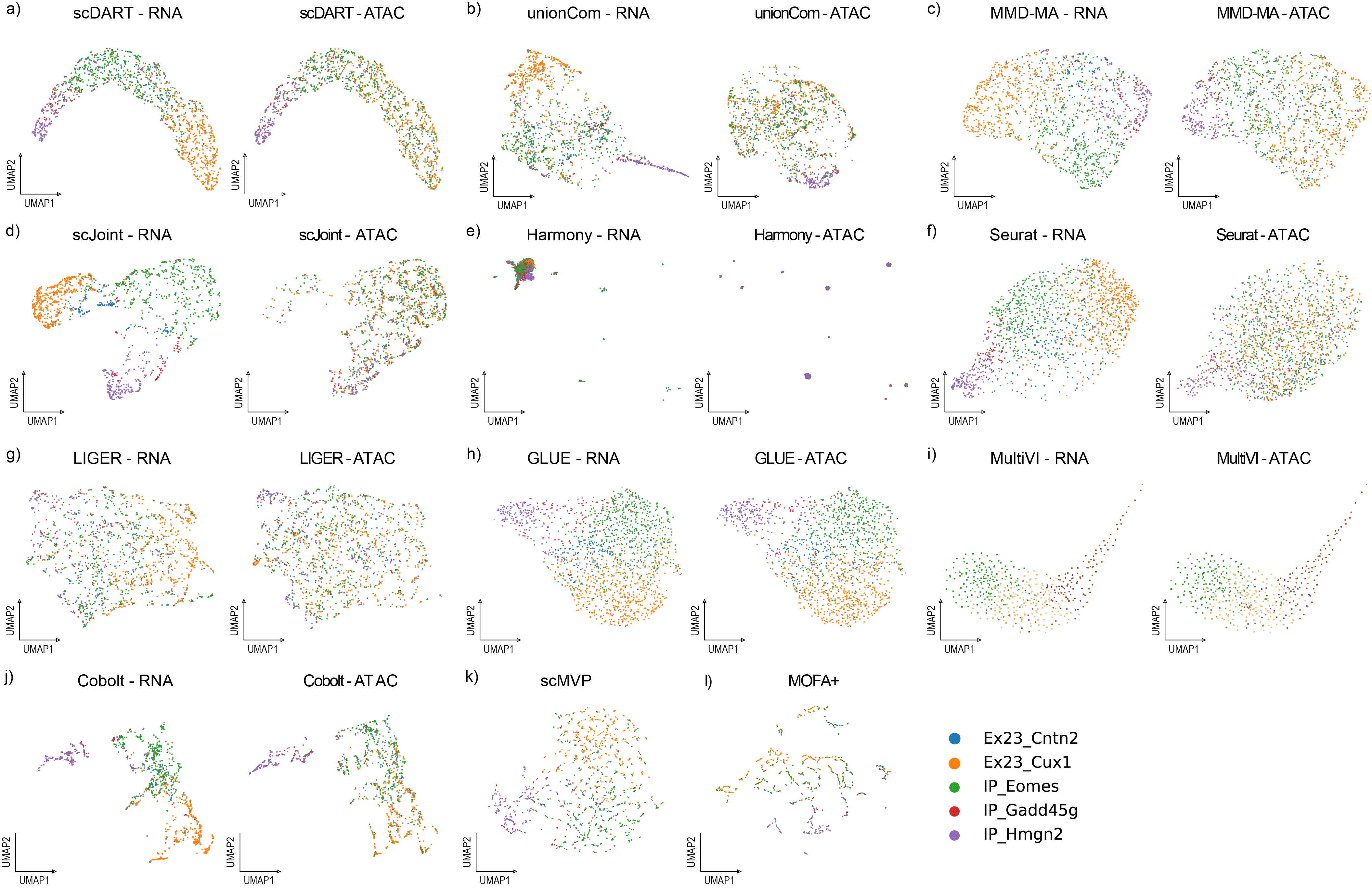


Fig. S4 | UMAP visualizations of the integrated cell embedding for Dataset-T, colored by cell types. We visualized the distributions of cells in scRNA-seq and scATAC-seq separately for unpaired integration methods, and visualized the distribution in a single figure for paired integration methods.
